# COVID-19 Vaccine Uptake among People with Spinal Cord Injury and Dysfunction in Ontario, Canada: A Population-Based Retrospective Cohort Study

**DOI:** 10.3390/healthcare12171799

**Published:** 2024-09-09

**Authors:** Angela Mei, Arrani Senthinathan, Swaleh Hussain, Mina Tadrous, Vanessa K. Noonan, Susan B. Jaglal, Rahim Moineddin, B. Catharine Craven, Sandra McKay, Lauren Cadel, John Shepherd, Karen Tu, Sara J. T. Guilcher

**Affiliations:** 1 Dalla Lana School of Public Health, University of Toronto, Toronto, ON M5S 1A1, Canada; 2 Leslie Dan Faculty of Pharmacy, University of Toronto, Toronto, ON M5S 3M2, Canada; 3 ICES, Toronto, ON M5T 3M6, Canada; 4 Women’s College Hospital Institute for Health Systems Solutions and Virtual Care, Women’s College Hospital, Toronto, ON M5S 1B2, Canada; 5 International Collaboration on Repair Discoveries, Vancouver, BC V5Z 1M9, Canada; 6 Praxis Spinal Cord Institute, Vancouver, BC V5Z 1M9, Canada; 7 Institute of Health Policy Management and Evaluation, University of Toronto, Toronto, ON M5T 3M6, Canada; 8 Department of Physical Therapy, Temerty Faculty of Medicine, University of Toronto, Toronto, ON M5S 1A8, Canada; 9 Rehabilitation Science Institute, University of Toronto, Toronto, ON M5G 1V7, Canada; 10 Department of Family and Community Medicine, University of Toronto, Toronto, ON M5G 1V7, Canada; 11 KITE (Knowledge Innovation Talent Everywhere), Toronto Rehabilitation Institute, University Health Network, Toronto, ON M4G 3V9, Canada; 12 Department of Medicine, Faculty of Medicine, University of Toronto, Toronto, ON M5S 1A8, Canada; 13 Spinal Cord Rehabilitation Program, Toronto Rehabilitation Institute, University Health Network, Toronto, ON M4G 3V9, Canada; 14 VHA Home HealthCare, Toronto, ON M4S 1V6, Canada; 15 North York General Hospital, Toronto, ON M2K 1E1, Canada; 16 Toronto Western Family Health Team, University Health Network, Toronto, ON M4G 3V9, Canada

**Keywords:** COVID-19 vaccine, disability, spinal cord injury and disease, health equity, sociodemographics, Ontario

## Abstract

Persons with disabilities experience numerous barriers to healthcare access including vaccine accessibility. The purpose of this study was to determine COVID-19 vaccine uptake in the spinal cord injury and disease (SCI/D) population of Ontario and identify potential factors influencing C OVID-19 vaccine uptake. This was a retrospective closed-cohort study using administrative health data on individuals with SCI/D of traumatic and non-traumatic causes to examine the monthly number of COVID-19 vaccine doses received between December 2020 and December 2023. Logistic regression analysis was used to examine the potential association between socio-demographic, clinical, and neighbourhood characteristics with initial COVID-19 vaccine receipt and booster dose uptake. By the end of the observation period in December 2023, 82.9% received the full two-dose coverage and 65.6% received at least one additional booster dose in a cohort of 3574 individuals with SCI/D. SCI/D individuals showed a comparable COVID-19 vaccine uptake percentage to the general population. Sociodemographic, clinical, and neighbourhood characteristics were associated with COVID-19 vaccine uptake in the SCI/D population, including age, type of injury, number of comorbidities, mental health history, and neighbourhood characteristics such as income. Further investigation is necessary to determine the causation effects of these relationships with vaccine uptake to address health equity concerns.

## 1. Introduction

As of May 2024, the World Health Organization (WHO) reported over 7 million COVID-19-related deaths globally since the virus onset [1]. In Canada, approximately 5 million COVID-19 cases and 59,000 COVID-19 related deaths were reported [2]. In addition to mortality, the COVID-19 infection rate contributed to short-term and long-term morbidity, including physical and psychological, on the affected person’s health and overall wellbeing [3]. Vulnerable populations, such as those with physical disabilities, older adults, females, those with lower income, and racialized groups, were particularly at risk of COVID-19 infection and severe health impacts [4,5,6].

The introduction of COVID-19 vaccines in December 2020 curbed the spread and mitigated further health impacts of the virus [7,8]. The COVID-19 virus continued to evolve with new mutations after the introduction of the initial recommended two-dose series of the COVID-19 vaccine [9,10,11,12,13,14]. The evolving mutations of the COVID-19 virus caused the failure of full viral protection with the initial vaccine series, and subsequent booster dosages were introduced to mitigate and contain the spread of COVID-19 variants [15,16]. The recommended target intervals of various booster dosages were dependent on the individual risk levels for adverse consequences and the regulations of the local public health units in Ontario, Canada [15,16]. COVID-19 vaccinations in Ontario were publicly funded and available through mass vaccination clinics, family physician offices, community pharmacies, and community outreach programs such as mobile clinics and home visits for high-risk groups. In the prior Canadian and global literature, the accessibility of the COVID-19 vaccine was not fully equitable across the population, with certain groups such as persons with lower income, those from racialized groups, and those with disabilities being particularly vulnerable to healthcare inequalities and intersectoral barriers to care [17,18,19].

Historically, populations with disabilities experience systemic barriers to accessing healthcare resources, especially during times of stress such as the COVID-19 pandemic [17,18]. A 2021 focus group study conducted in Manitoba, Canada, reported that individuals with disabilities were unequally challenged with barriers in receiving COVID-19 vaccine-related information, booking vaccination appointments, and physically accessing the vaccination sites [20]. Beyond physical disabilities, research has shown that a history of mental health conditions impacts the uptake of the COVID-19 vaccine as well [21,22,23]. Within the disabled population, the spinal cord injury and disease (SCI/D) population was particularly vulnerable to healthcare inequalities and challenges with vaccine access due to mobility barriers, comorbidities, and resource limitations [24,25,26,27]. Furthermore, many individuals with SCI/D also face mental health challenges, which were exacerbated during the COVID-19 pandemic and may have impacted their vaccine uptake [28,29]. Ensuring the uptake of the COVID-19 vaccine in individuals with SCI/D is important as they are prone to serious respiratory infections, including pneumonia, which is one of the leading causes of mortality in the SCI/D population [28,30,31,32]. A previous cross-sectional survey on community-dwelling individuals with SCI/D displayed lower influenza vaccine uptake compared to the guideline recommendation [33]. These factors may disproportionally impact certain subgroups of individuals with SCI/D with increasing vulnerability to healthcare inequalities and severe healthcare consequences. No population-based administrative health data research has been conducted in North America describing the percentage of COVID-19 vaccine uptake in the SCI/D population or other populations with physical disabilities living in the community. This knowledge gap has implications for public health, as individuals with certain specific disabilities are at increased risk for severe COVID-19 outcomes [26,34]. Investigating the COVID-19 vaccine uptake in the SCI/D population across different subgroups can provide insight into health service equity and access to guide policy and development. As such, detailed information on factors influencing vaccine uptake within the SCI/D population are needed. The purpose of this study was to investigate the rates of COVID-19 vaccination in the SCI/D population and to identify the impact of sociodemographic, clinical (e.g., sex, age, comorbidities, medications dispensed), and neighbourhood characteristics on vaccine uptake.

## 2. Methods

This was a retrospective closed-cohort study using linked health administrative databases from ICES in Ontario, Canada. ICES is an independent, non-profit research institute funded by an annual grant from the Ontario Ministry of Health and the Ministry of Long-Term Care. As a prescribed entity under Ontario’s privacy legislation, ICES is authorized to collect and use healthcare data for the purposes of health system analysis, evaluation, and decision support. Secure access to these data is governed by policies and procedures approved by the Information and Privacy Commissioner of Ontario. The use of the data in this project is authorized under section 45 of Ontario’s Personal Health Information Protection Act (PHIPA) and is approved by the ICES privacy office; as such, it does not require further review by a research ethics board.

### 2.1. Data Source

Data for this study were obtained from ICES. The Discharge Abstract Database (DAD) and the National Rehabilitation System (NRS) database were both used to identify individuals who would comprise the cohort for this study. The COVID-19 vaccination information system of Ontario (COVaxON) database was used to obtain information related to COVID-19 vaccination. COVID-19 vaccines and boosters are publicly funded in Canada and are available without additional costs to individuals.

Patient characteristics collected included sociodemographics (age, sex, and postal code) from the Registered Persons Database (RPDB). Individuals’ postal codes from the RPDB were used to identify neighbourhood characteristics based on rurality and income level stratification. The number of medications dispensed was obtained from the Ontario Drug Benefits Database (ODB). The ODB provides information on medications dispensed for those under the government’s public drug programs including persons aged 65 years or older in Ontario, residents of long-term care homes, individuals with no private drug insurances, and recipients of professional home services and social assistances. A history of mental health concerns was identified in the DAD and the Ontario Health Insurance Plan (OHIP) database based on flags in a multimorbidity macro for mood disorders and other mental health conditions [35]. The presence of either or both conditions indicated that an individual had a history of mental health conditions or a mental health diagnosis.

The Ontario Marginalization Index (ON-Marg) was used to identify and report the following neighbourhood characteristics as quintiles: households and dwellings, age and labour force, and racialized and newcomer populations. The households and dwellings quintiles measure the types and density of residential accommodations, and certain family structure characteristics, such as number of individuals living alone and number of unowned dwellings in each neighbourhood. Age and labour force quintiles include indicators to describe the dependency ratio (seniors and children within the population from 15 to 64 years old) and the number of individuals not participating in the labour force within a neighbourhood. The racialized and newcomer population quintiles indicate the number of recent immigrants and those who self-identify as a ‘visible minority’ within a neighbourhood.

The Johns Hopkins ACG^®^ System Version 10 was used to categorize individuals by resource utilization bands (RUBs). RUBs evaluate the overall morbidity level of the individuals and are indicators of expected health-related resource utilization. The RUB scale ranges from 0 to 5, with higher values associated with higher utilization levels. The SCI/D population has higher comorbidities and RUBs are a useful indicator to address the interactions among the comorbidities [36].

### 2.2. Cohort

The cohort used for this study was a sub-cohort from a previously developed study investigating healthcare utilization during the pandemic for SCI/D [26]. The cohort consisted of individuals with traumatic and non-traumatic causes of SCI/D. Individuals with a traumatic spinal cord injury (TSCI) were identified from the DAD based on first admission and discharge from acute hospitalization between 1 April 2004 and 28 February 2014. The cohort cut-off on 28 February 2014 ensured that individuals were community-dwelling for at least 5 years before the start of the COVID-19 pandemic. Individuals with non-traumatic spinal cord dysfunction (NTSCD) were identified from the NRS database based on first admission and discharge from inpatient rehabilitation between 1 April 2004 and 28 February 2014. Only individuals living in the community per a hospital discharge summary since 2014 and who were alive during the observation window from December 2020 to December 2023 per discharge record were included in the study. The number of monthly COVID-19 vaccine doses were identified from December 2020 to December 2023 in a community-dwelling SCI/D cohort.

### 2.3. Outcomes

The COVaxON database records all COVID-19 vaccines administered in Ontario in its real-time centralized vaccine information system, which also collected information on the date and location of administration, product type, and dose number. We extracted records of vaccination for the SCI/D cohort from December 2020 to December 2023. To evaluate the uptake of the vaccine, we calculated the percent of individuals within the cohort who received 0 to 5 doses by monthly evaluation. Vaccination full coverage was classified as at least 2 doses, with 3+ doses classified as additional booster doses [9,10,11,12,13,14,16]. Vaccination uptake was stratified and evaluated by the following sociodemographic characteristics: injury type (TSCI vs. NTSCD), sex (males vs. females), and age (under 65 years vs. 65 years and over).

### 2.4. Statistical Analysis

Means, standard deviations, and proportions were used to describe the demographic characteristics for the SCI/D cohort stratified by vaccine doses (0 doses, 1 dose only, 2 doses only, and 3+ doses). Two multivariable logistic regression models were conducted to determine if patient demographic, clinical, and neighbourhood characteristics were associated with COVID-19 vaccination coverage by December 2023. An assessment of multicollinearity was conducted and factors that exhibited multicollinearity were not modeled together in the regression analyses. The first model examined parameters related to the uptake of the initial full vaccination coverage of two doses (zero doses compared to a minimum of two doses) [9,10,11,12,13,14,16]. The second model examined factors associated with receiving the booster dose in addition to the initial two-dose series (2 doses only compared to 3 or more doses). Missing data were merged with the highest category. The Hosmer–Lemeshow test was conducted for each model to determine goodness of fit.

## 3. Results

**Figure 1:**
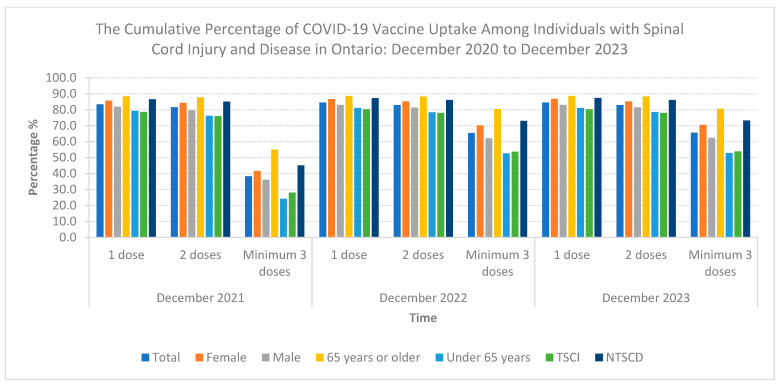
The cumulative percentage of COVID-19 vaccine uptake among individuals with spinal cord injury and disease in Ontario: December 2020 to December 2023. Graphical representation of the cumulative percentage of COVID-19 uptake (1 dose, 2 doses, and 3 or more doses) at the timepoints of December 2021, December 2022, and December 2023, stratified by sex, age and type of spinal cord injury and disease (traumatic spinal cord injury (TSCI) and non-traumatic spinal cord dysfunction (NTSCD)). Individuals of 65 years or older or with NTSCD have a higher percentage of COVID-19 vaccine uptake. See Table 2 for specific percentage values.

**Figure 2:**
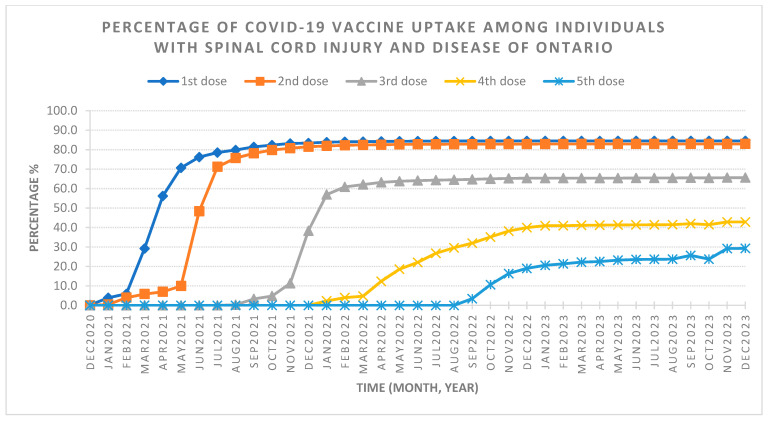
Full cohort of COVID-19 vaccine uptake among individuals with spinal cord injury and dysfunction (SCI/D) living in the community setting of Ontario. The graph displays the percentage of COVID-19 vaccine uptake from December 2020 to December 2023 for the Ontario spinal cord injury and disease population living in the community setting. At the end of the retrospective cohort study, individuals can receive up to a maximum of five doses of the COVID-19 vaccine according to local public health guidelines. Total number of individuals in the cohort is 3574. At the end of December 2023, total percentage of COVID-19 vaccine is 84.5% for first dose, 82.9% for second dose, 65.6% for third dose, 42.9% for fourth dose, and 29.3% for fifth dose.

**Figure 3:**
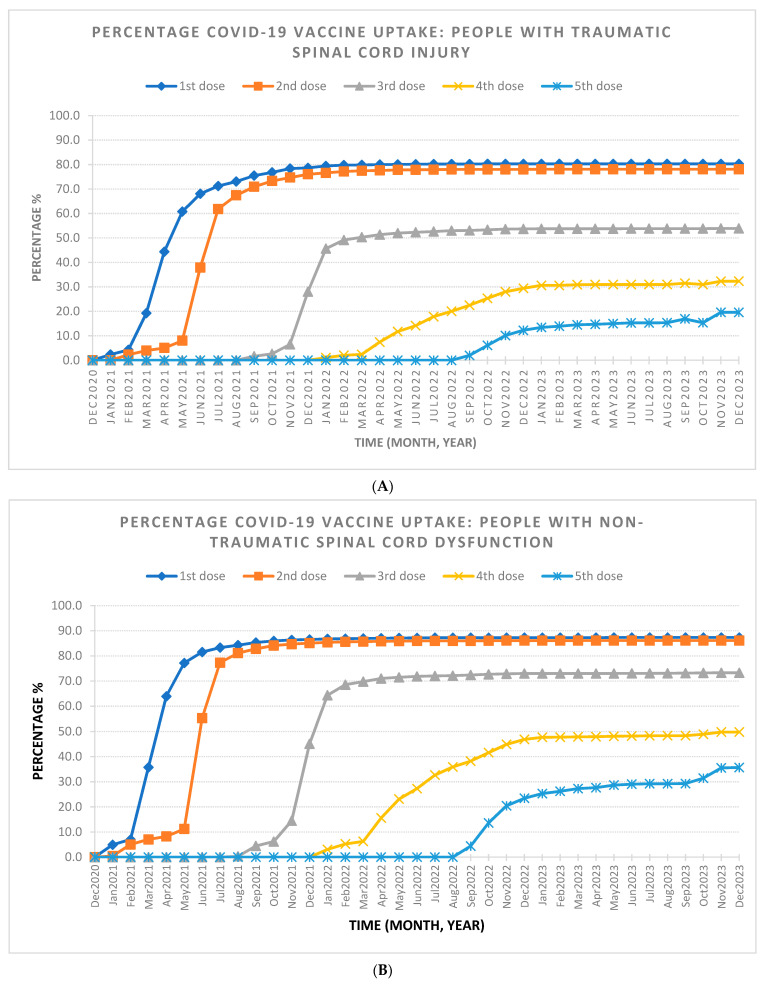
The percentage of COVID-19 vaccine uptake from December 2020 to December 2023 for people with traumatic and non-traumatic spinal cord-injuries and dysfunction living in the community setting. (**A**) The percentage of COVID-19 vaccine uptake for people with traumatic spinal cord-injuries (TSCI). Total population in the TSCI group is 1414. At the end of December 2023, percentage of COVID-19 vaccine uptake is 80.3% for first dose, 78.1% for second dose, 53.9% for third dose, 32.3% for fourth dose, and 19.6% for fifth dose. (**B**) The percentage of COVID-19 vaccine uptake for people with non-traumatic spinal cord dysfunction(NTSCD). Total population in the NTSCD group is 2142. At the end of December 2023, percentage of COVID-19 vaccine uptake is 87.3% for first dose, 86.1% for second dose, 73.3% for third dose, 49.8% for fourth dose, and 35.7% for fifth dose.

**Figure 4:**
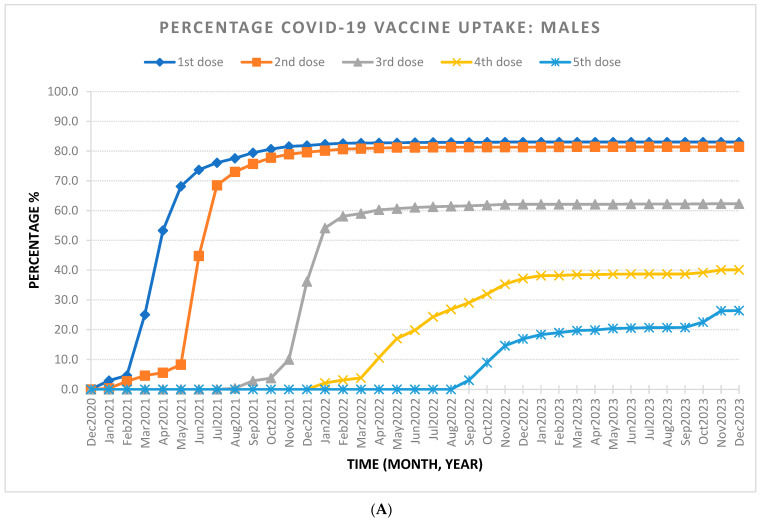

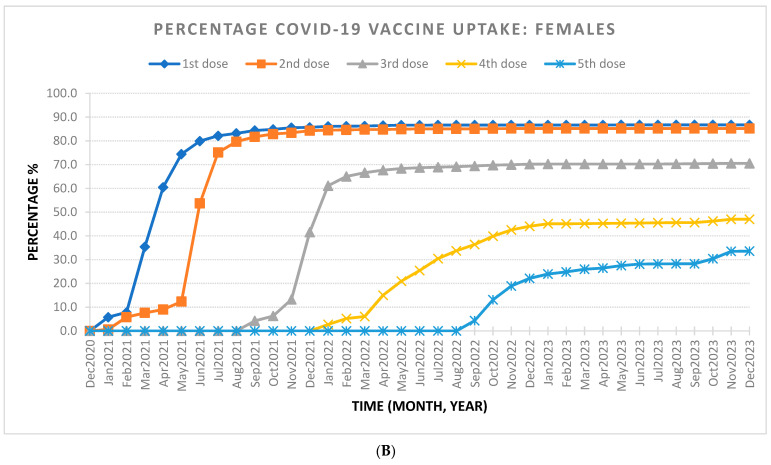
The percentage of COVID-19 vaccine uptake from December 2020 to December 2023 based on gender for people with traumatic and non-traumatic spinal cord-injuries and dysfunction living in the community setting. (**A**) The percentage of COVID-19 vaccine uptake for males. Total male population is 2142. At the end of December 2023, percentage of COVID-19 vaccine uptake is 83.1% for first dose, 81.4% for second dose, 62.3% for third dose, 40.1% for fourth dose, and 26.4% for fifth dose. (**B**) The percentage of COVID-19 vaccine uptake for females. Total female population is 1432. At the end of December 2023, percentage of COVID-19 vaccine uptake is 86.7% for first dose, 85.2% for second dose, 70.5% for third dose, 47.0% for fourth dose, and 33.6% for fifth dose.

**Figure 5:**
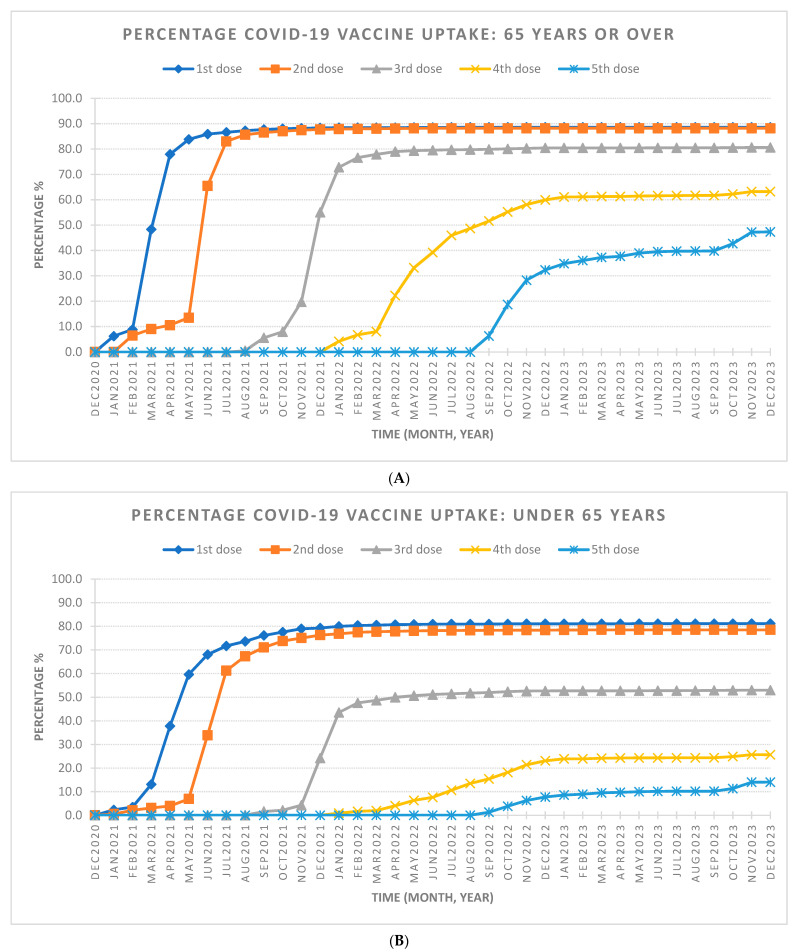
The percentage of COVID-19 vaccine uptake from December 2020 to December 2023 based on age for people with traumatic and non-traumatic spinal cord-injuries and dysfunction living in the community setting. (**A**) The percentage of COVID-19 vaccine uptake for 65 years old and over. Total 65 years old and over population is 1637. At the end of December 2023, percentage of COVID-19 vaccine uptake is 88.6% for first dose, 88.2% for second dose, 80.6% for third dose, 63.2% for fourth dose, and 47.3% for fifth dose. (**B**) The percentage of COVID-19 vaccine uptake for under 65 years old. Total under 65 years old population in the spinal cord-injured group is 1937. At the end of December 2023, percentage of COVID-19 vaccine uptake is 81.1% for first dose, 78.5% for second dose, 52.9% for third dose, 25.7% for fourth dose, and 14.0% for fifth dose.

The final SCI/D cohort included 3574 individuals, with 60.4% diagnosed with NTSCD and 39.6% diagnosed with TSCI. Most of the cohort was male (59.9%) and under 65 years old (54.2%). Table 1 outlines the demographic characteristics stratified by dose uptake. By the end of the observation period in December 2023, 82.9% of individuals reached full vaccine coverage (two doses), with 65.6% receiving additional doses (see Table 2 and Figure 1). Saturation of full coverage was reached by December 2021 and saturation of additional doses was reached by December 2022 (Figure 2). The stratification of COVID-19 vaccine uptake by SCI/D injury type showed a higher percentage of COVID-19 vaccine uptake by individuals with NTSCD compared to TSCI (Figure 3). Figure 4 shows higher COVID-19 vaccine uptake in females compared to males. Stratification by age showed higher COVID-19 vaccine uptake in individuals of 65 years and older compared to under 65 years old (Figure 5).

### 3.1. Regression Results

#### 3.1.1. Model 1 (Table 3): Evaluation of Full COVID-19 Vaccine Coverage (Minimum Two Doses)

SCI/D individuals 44 years old and under were significantly less likely to receive full coverage compared to individuals 65 years and older (OR: 0.64, 95% CI: 0.46 to 0.90, *p*-value: 0.01). Individuals with NTSCD were significantly more likely to receive full coverage compared to TSCI individuals (OR: 1.29, 95% CI: 1.02 to 1.64, *p*-value: 0.03). Individuals with a resource utilization band of zero compared to band five were significantly less likely to receive full coverage (OR: 0.09, 95% CI: 0.06 to 0.13, *p*-value: <0.01). Individuals with no medications dispensed were significantly less likely to receive full coverage compared to individuals using greater or equal to 20 medications (OR: 0.43, 95% CI: 0.26 to 0.70, *p*-value: <0.01). Individuals with three to four medical conditions were significantly less likely to receive full coverage compared to individuals with five or more diagnosed health conditions (OR: 0.66, 95% CI: 0.49 to 0.89, *p*-value: <0.01). Sex and history of mental conditions were not related to the uptake of the COVID-19 vaccine full coverage.

For neighbourhood characteristics, lower neighbourhood income quintiles 1 (OR: 0.41, 95% CI: 0.26 to 0.64), 2 (OR: 0.38, 95% CI: 0.25 to 0.57), and 3 (OR: 0.56, 95% CI: 0.37 to 0.83) were significantly less likely to receive the full coverage vaccine compared to the highest income quintile 5. Individuals from the neighbourhood households and dwellings quintile 3 were significantly more likely to receive full coverage compared to quintile 5 (OR: 1.50, 95% CI 1.03 to 2.18, *p*-value 0.04). Individuals from neighbourhoods of lower age and labour force quintiles 1 (OR: 0.65, 95% CI: 0.45 to 0.92, *p*-value: 0.01), 3 (OR: 0.69, 95% CI: 0.49 to 0.98, *p*-value: 0.04), and 4 (OR: 0.69, 95% CI: 0.49 to 0.96, *p*-value: 0.03) were significantly less likely to receive full coverage compared to quintile 5. Racialized and newcomer population quintiles of neighbourhood did not significantly affect COVID-19 vaccine uptake.

#### 3.1.2. Model 2 (Table 3): Evaluating COVID-19 Booster Dose Uptake

Younger individuals (44 years and under (OR: 0.21, 95% CI: 0.15 to 0.29, *p*-value: <0.01); 45 to 64 years (OR: 0.40, 95% CI: 0.31 to 0.54, *p*-value: <0.01)) were significantly less likely to receive boosters compared to individuals 65 years and older. Individuals with NTSCD were significantly more likely to receive boosters compared to individuals with TSCI (OR: 1.54, 95% CI: 1.24 to 1.91, *p*-value: <0.01). Individuals with a lower resource utilization band (1 (OR: 0.34, 95% CI: 0.16 to 0.72, *p*-value: <0.01), 2 (OR: 0.49, 95% CI: 0.32 to 0.76, *p*-value: <0.01), 3 (OR: 0.61, 95% CI: 0.45 to 0.82, *p*-value: <0.01), and 4 (OR: 0.68, 95% CI: 0.49 to 0.95, *p*-value: 0.02)) were significantly less likely to receive boosters compared to band 5. Individuals with no medication (OR: 0.46, 95% CI: 0.30 to 0.72, *p*-value: <0.01) and lower medication use (10 to 19 medications (OR: 0.62, 95% CI: 0.41 to 0.94, *p*-value: 0.02)) were less likely to receive boosters compared to individuals who required 20 or more medications. Compared to those diagnosed with a mental health condition, individuals with no prior history of mental health condition diagnosis were significantly more likely to receive the COVID-19 vaccine booster (OR: 1.47, 95% CI: 1.14 to 1.90, *p*-value: <0.01).

For neighbourhood characteristics, lower income quintiles (1 (OR: 0.39, 95% CI: 0.26 to 0.59, *p*-value: <0.01); 2 (OR: 0.67, 95% CI: 0.47 to 0.97, *p*-value: 0.03)) were less likely to receive boosters compared to the highest income quintile 5. Individuals from lower households and dwellings quintiles (1 (OR: 0.48, 95% CI: 0.32 to 0.72, *p*-value: <0.01), 2 (OR: 0.49, 95% CI: 0.33 to 0.71, *p*-value: <0.01), 3 (OR: 0.52, 95% CI: 0.36 to 0.75, *p*-value: <0.01), and 4 (OR: 0.51, 95% CI: 0.38 to 0.70, *p*-value: <0.01)) were significantly more likely to receive boosters compared to quintile 5. Individuals from age and labour force quintile 1 (OR: 0.67, 95% CI: 0.48 to 0.92, *p*-value: 0.02) were less likely to receive boosters compared to quintile 5. Parameters showing no statistical significance on the impact of COVID-19 booster vaccine uptake included sex, racialized and newcomer population quintile, and the number of health conditions.

## 4. Discussion

This population-based study investigated the rates of COVID-19 vaccination in the Ontario SCI/D population and identified the impact of sociodemographic, clinical (i.e., sex, age, comorbidities, medications dispensed), and neighbourhood characteristics on vaccine uptake. First, over 80% of the community-dwelling SCI/D population received full coverage (minimum of two doses) of the COVID-19 vaccine within one year after vaccine availability, from December 2020 to 2021. Second, over 65% of the community-dwelling SCI/D population also received an additional booster by December 2022. Importantly, certain sociodemographic, clinical, and neighbourhood characteristics impacted the likelihood of receiving full coverage, as well as boosters.

The Ontario SCI/D population’s COVID-19 vaccine uptake (84.5% for one dose) is comparable to that of the general population in Canada (84.7%) and slightly higher than in the general population of Ontario (80.9%) [37]. Significantly, the Ontario SCI/D population vaccine uptake is higher compared to the internationally reported COVID-19 vaccine uptake. A previous cross-sectional study in Thailand reported the uptake of the COVID-19 vaccine in the SCI/D population comparable to the general population for one (77% vs. 78.2%) and two doses (70% vs. 71.9%), respectively, and a lower uptake of the booster dose (20% vs. 31.3%) [24]. A cross-sectional study in Kenya reported a COVID-19 vaccine uptake of 65.7% in persons with disabilities and the vaccination barriers of cost of transportation, lack of mobile clinics, lack of access to reliable vaccine information, the belief in COVID-19 infection, and marital status [25]. In Ontario, the presence of mobile clinics, home visits, and community-accessible vaccination possibly supported its higher COVID-19 vaccine uptake compared to Thailand and Kenya. In the Ontario SCI/D population, the decrease in booster dose uptake (65.6%) in comparison with the uptake of initial two-dose series (82.9%) could be due to a decline in vaccine interests and changes in vaccine-service availability in the community setting (i.e., reduced mass vaccination clinics, mobile clinics, etc.), considering these services mostly prevailed during the vaccination initiation phase in 2021 [15,16]. Vaccination initiatives at local community sites can better accommodate individuals with disabilities and possibly support the increase in COVID-19 vaccine uptake in Ontario compared to global trends. To compare the COVID-19 vaccine uptake in other populations, a multiple center study in Germany reported comparable COVID-19 vaccine uptake between the multiple sclerosis population with the general population in the uptake of the first (84.5% and 78.0%) and second dose of the COVID-19 vaccine (78.2% vs. 76.4%) [38]. These results suggested that the presence of disability alone may not significantly impact the uptake of the COVID-19 vaccine compared to the general population.

The previous literature showed the association between COVID-19 vaccine uptake and various factors including physical barriers, mobility limitations, vaccine hesitancy, societal attitudes, logistical difficulties, information accessibility, and socioeconomic factors [18,24,25,39,40,41,42]. Our study found the younger SCI/D populations were less likely to obtain COVID-19 vaccines compared to the older population of 65 years or older. This finding is consistent with the investigation of COVID-19 vaccine hesitancy in the United States, which reported younger adults had higher vaccine hesitancy compared to the older population [43]. Younger populations may be more concerned about the side effects of the vaccine compared to the outcomes of the COVID-19 infection because they have fewer comorbidities causing severe outcomes [44,45,46]. Older individuals may be at greater risk of experiencing complications and severe outcomes if infected with the COVID-19 virus [46]; as such, these individuals may have been more diligent with ensuring they are up-to-date with COVID-19 vaccination guidelines.

Surprisingly, sex was not associated with the uptake of full COVID-19 vaccine coverage or the booster dose from the multivariable logistic regression analysis. A previous systematic review and exploratory analysis reported higher vaccine hesitancy rates among females, with vaccine safety being the major concern [43,47]. However, a recent scoping review showed that vaccine hesitancy does not necessarily equate with the behaviour of absence of vaccine uptake [48]. Canadian national statistics showed higher COVID-19 vaccine uptake of at least one dose and per recommendation in females (84.7% and 21.3%) compared to males (79.7%, and 18.7%) for those of five years and older [37]. Our study results showed similar findings of full coverage vaccine uptake in female (85.2%) compared to males (81.4%). The fifth dose of the COVID-19 vaccine is the latest recommended booster dose at the end of the study, with 33.6% uptake in females, and 26.4% in males, showing a slightly higher vaccine uptake compared to the general population. However, after correcting for sociodemographic factors, there were no statistical significance findings between vaccine uptake and sex. This suggests that there are other sociodemographic factors impacting vaccine uptake, which require further investigation.

With respect to injury type, individuals with NTSCD were more likely to obtain the COVID-19 vaccine compared to individuals with TSCI. This finding differs from the previous literature report from Thailand that individuals with NTSCD were less likely to receive a COVID-19 vaccine due to speculated concerns about adverse reactions toward the vaccine [24]. Healthcare resource utilization, the number of medications dispensed, the number of health conditions, and mental health status also seemed to impact vaccination uptake at varying levels. Individuals with three to four pre-existing medical conditions were less likely to receive full COVID-19 vaccine coverage compared to those who were diagnosed with five or more health conditions. Interestingly the diagnosis of mental health conditions did not impact the initial COVID-19 vaccine uptake, and the number of health conditions did not impact the uptake of the COVID-19 booster dose. RUB usage, an indicator for overall morbidity and healthcare resource utilization, was associated with both initial vaccine uptake and booster dose uptake and could indicate the potential of those with a higher disease burden to be more inclined for vaccination due to the potential concerns about severe health impacts after COVID-19 infection [49].

Finally, neighbourhood characteristics were found to be important factors associated with the COVID-19 vaccine uptake in this study. Certain neighbourhoods may have barriers to vaccine accessibility and dissemination. Individuals from lower-income neighbourhoods were less likely to receive the full-coverage COVID-19 vaccine or the booster dose. Households and dwellings composition, as well as age and labour factors of a neighbourhood, correlated with receiving full COVID-19 vaccine coverage and booster doses. Lower residency stability decreased the booster dose uptake but did not associate with the uptake of the full COVID-19 vaccine series. A low dependency ratio (age and labour force quintile) suggested that a higher proportion of working adults compared to children and seniors showed decreased COVID-19 vaccine and booster dose uptake. Neighbourhood characteristics were associated with vaccine uptake, but further investigations are needed to understand the implication of these findings.

### 4.1. Limitations

This is a closed-cohort study focused on a community-dwelling population with SCI/D diagnoses in Ontario for at least 5 years prior to the start of the COVID-19 pandemic and receiving rehabilitation. Individuals with SCI/D living in the community for less than 5 years prior to the start of the COVID-19 pandemic and individuals who passed away before the end of the cohort study at the end of December 2023 were excluded from this study. This study is limited by the extent and type of data available in the databases with the secondary use of health administrative data. It is possible that individuals in the cohort may have received COVID-19 vaccines outside of Ontario, which would not be captured in the COVaxON database. However, due to travel restrictions during this time, as well as limited mobility in our population, this is unlikely. This study assessed COVID-19 vaccine uptake based on the number of dosages received and did not clinically evaluate the type of vaccination and exact interval of vaccination to determine whether the population studied was vaccinated per recommendation of the Public Health Unit. The ODB only captures prescribed medications that are dispensed for individuals 65 years or older and those who receive social assistance. Thus, medications covered by private insurance, dispensed in clinics or hospitals, and prescribed to individuals younger than 65 years old not on the Ontario public drug insurance plan were not captured in this study. Additionally, over-the-counter products, natural health products, and supplements are not captured in this study. Thus, the record of no medications dispensed should be interpreted with caution. Despite this, number of health conditions and the health-related burden are captured with the ICES data and may be a good estimate for the disease burden. Due to limitations in cohort size, parameters of rurality and the first dose were not included in the regression analyses. The results of our study reflected the SCI/D population in Ontario only. As such, inferences about other health systems and regions should be made with caution.

### 4.2. Future Directions

For future pandemic response and preventative-measure planning, further investigations on the reasons for vaccine hesitancy and increased vaccination compliance need to be conducted. Potential concerns include vaccine fatigue (five total doses, and three booster doses introduced as of December 2023), public trust, reduction in mobile clinics, and vaccination sites with booster doses compared to 2021 when vaccines were first introduced. With the increasing number of booster dosages, barriers to vaccinations become more evident with a potential increase in vaccine fatigue within the population. In the future, targeted health policies and strategies need to be implemented with the consideration of the sociodemographic factors driving vaccine hesitancy and reducing barriers to healthcare access for vulnerable populations. For instance, neighbourhood characteristics were found to be correlated with the uptake of the COVID-19 vaccine and booster doses, and further research may be necessary to determine the relationship between the neighbourhood potential for health-resource limitations. Further mixed-method investigations are necessary to understand individual, community, and health-system factors impacting vaccination delivery and uptake. Considering that our study uses existing health administrative data, we did not evaluate marital status or individual perspectives on vaccines or the COVID-19 infection or the education levels of the cohort population. Further studies are needed to investigate these parameters.

## 5. Conclusions

Individuals with physical disabilities, such as SCI/D, frequently experience barriers to healthcare access, and addressing the intersection between disability and other disparities in vaccine uptake is critical to highlighting systemic inequities. This study evaluated the uptake of the COVID-19 vaccine within the SCI/D population and examined the parameters potentially affecting vaccine uptake such as clinical or sociodemographic characteristics (i.e., TSCI and NTSCD, sex, age, and neighbourhood characteristics). Our study found the rate of COVID-19 vaccine uptake in the SCI/D population was comparable to the general population. The comparable initial uptake of the COVID-19 vaccine and the subsequent booster doses between individuals with SCI/D and the general population in Ontario suggested the lack of significant differences with COVID-19 vaccine access associated with the sole diagnosis of SCI/D. The regression analyses showed variability in COVID-19 vaccine uptake amongst the subpopulations of SCI/D. Within the SCI/D subpopulations, individuals diagnosed with NTSCD were statistically more likely to receive the COVID-19 vaccines compared to TSCI. The SCI/D subpopulation analysis also showed that persons less than 44 years old and from neighbourhoods with lower incomes and a low dependency ratio exhibited lower uptake of the initial COVID-19 vaccine series and the booster doses. Community-dwelling SCI/D individuals with the characteristic of lower predicted resource utilization (low RUB quintile) and from neighbourhoods with less residential stability exhibited a lower uptake of the COVID-19 booster dose. The findings from this study provide opportunities for public health policy to modify the vaccination programs with increased awareness and sensitivity to sociodemographic data. This is particularly valuable in situations where multiple vaccination doses or boosters are required to achieve the targeted preventative health goals and the closure of vital community-based access points (i.e., mobile clinics and home visits) may disproportionately impact vulnerable populations.

The immediate threat of the COVID-19 virus has significantly diminished, mainly due to the global efforts of COVID-19 vaccination [7,8]. It is important to continue evaluating vaccine uptake in persons with disabilities to inform public health preparedness for future pandemics or infectious disease outbreaks. Ontario offered outreach programs for individuals at high risk for COVID-19 infection complications and living at home, which may increase the percentage of uptake of the COVID-19 vaccine within the community-dwelling SCI/D population [16]. Lessons learned from the COVID-19 pandemic can help to develop more effective strategies for reaching and vaccinating vulnerable populations in future health emergencies or seasonal flu outbreaks. The findings of this study can help inform policy, optimize interventions, and ultimately foster better health outcomes for people with disabilities in the context of the ongoing pandemic and future health emergencies.

## Figures and Tables

**Table 1:** Demographic table for the spinal cord injury and disease (SCI/D) population living in the community setting from December 2020 to December 2023 in Ontario, Canada.

	SCI/D Cohort		No Dose		2 Doses *		3+ Doses	
	(N = 3574)		N = 553 (15.5%)		N = 676 (18.9%)		N = 2345 (65.6%)	
Characteristics	n	%	n	%	n	%	n	%
Sex								
Female	1432	40.1	190	13.3	232	16.2	1010	70.5
Male	2142	59.9	363	16.9	444	20.7	1335	62.3
Age								
Under 65 years old	1937	54.2	366	18.9	546	28.2	1025	52.9
65 years and over	1637	45.8	187	11.4	130	7.9	1320	80.6
Injury Type								
Non-Traumatic Spinal Cord Dysfunction (NTSCD)	2160	60.4	274	12.7	303	14.0	1583	73.3
Traumatic Spinal Cord Injury (TSCI)	1414	39.6	279	19.7	373	26.4	762	53.9
Income Quintile **								
Q1 (low)	856	24.0	151	17.6	192	22.4	513	59.9
Q2	799	22.4	148	18.5	136	17.0	515	64.5
Q3	655	18.3	97	14.8	120	18.3	438	66.9
Q4	606	17.0	85	14.0	116	19.1	405	66.8
Q5 (high)	658	18.4	72	10.9	112	17.0	474	72.0
Racialized and Newcomer Population Quintile								
1	673	18.8	108	16.0	144	21.4	421	62.6
2	693	19.4	82	11.8	128	18.5	483	69.7
3	621	17.4	105	16.9	99	15.9	417	67.1
4	742	20.8	112	15.1	119	16.0	511	68.9
5	802	22.4	139	17.3	178	22.2	485	60.5
missing	43	1.2	7	16.3	8	18.6	28	65.1
Households and Dwellings Quintile								
1	538	15.1	80	14.9	109	20.3	349	64.9
2	607	17.0	89	14.7	121	19.9	397	65.4
3	619	17.3	75	12.1	129	20.8	415	67.0
4	732	20.5	117	16.0	154	21.0	461	63.0
5	1035	29.0	185	17.9	155	15.0	695	67.1
missing	43	1.2	7	16.3	8	18.6	28	65.1
Age and Labour Force Quintile								
1	650	18.2	121	18.6	150	23.1	379	58.3
2	654	18.3	108	16.5	133	20.3	413	63.1
3	620	17.3	101	16.3	122	19.7	397	64.0
4	645	18.0	99	15.3	124	19.2	422	65.4
5	962	26.9	117	12.2	139	14.4	706	73.4
missing	43	1.2	7	16.3	8	18.6	28	65.1
Resource Utilization Bands (RUBs)								
0 (low)	269	7.5	186	69.1	28	10.4	55	20.4
1	51	1.4	10	19.6	20	39.2	21	41.2
2	267	7.5	50	18.7	82	30.7	135	50.6
3	1457	40.8	170	11.7	329	22.6	958	65.8
4	686	19.2	58	8.5	113	16.5	515	75.1
5 (high)	844	23.6	79	9.4	104	12.3	661	78.3
Counts for Unique Medications Mean 8.5 (SD = 9.1)								
0	1071	30.0	314	29.3	306	28.6	451	42.1
1–9	1070	29.9	132	12.3	197	18.4	741	69.3
10–19	957	26.8	77	8.0	128	13.4	752	78.6
≥20	476	13.3	30	6.3	45	9.5	401	84.2
Previous Diagnosis of Mental Condition(s)								
Yes	2568	71.9	363	14.1	495	19.3	1710	66.6
No	1006	28.1	190	18.9	181	18.0	635	63.1
Number of Health Conditions								
0 to 2	1019	28.5	234	23.0	275	27.0	510	50.0
3 to 4	1174	32.8	194	16.5	232	19.8	748	63.7
5+	1381	38.6	125	9.1	169	12.2	1087	78.7

* Due to the small sample size, individuals with one dose of the COVID-19 vaccine only are merged with the individuals with two doses of the COVID-19 vaccine to prevent small cells from re-calculation and identification. Less than 2% of the full cohort had one dose. ** To avoid small cells from being re-calculated, missing data were suppressed in the income quintile with the highest percentage.

**Table 2:** The cumulative percentage of COVID-19 vaccine uptake among individuals with spinal cord injury and disease in Ontario: December 2020 to December 2023. The cumulative percentage of COVID-19 uptake (1 dose, 2 doses, and 3 or more doses) at the timepoints of December 2021, December 2022, and December 2023, stratified by sex, age and type of spinal cord injury and disease (traumatic spinal cord injury (TSCI) and non-traumatic spinal cord dysfunction (NTSCD)).

	December 2021			December 2022			December 2023		
	1 Dose	2 Doses	3 Doses	1 Dose	2 Doses	Minimal 3 Doses	1 Dose	2 Doses	Minimal 3 Doses
Total	83.4	81.5	38.3	84.5	82.9	65.4	84.5	82.9	65.6
Female	85.7	84.3	41.6	86.7	85.2	70.2	86.7	85.2	70.5
Male	81.9	79.6	36.2	83.1	81.3	62.1	83.1	81.4	62.3
65 or over	88.3	87.7	55.0	88.6	88.2	80.5	88.6	88.2	80.6
Under 65	79.2	76.3	24.2	81.1	78.4	52.6	81.1	78.5	52.9
TSCI	78.6	76.0	28.0	80.3	78.0	53.7	80.3	78.1	53.9
NTSCD	86.5	85.1	45.1	87.3	86.1	73.0	87.3	86.1	73.3

**Table 3:** Multivariable logistic regression models of potential characteristics influencing the uptake of full COVID-19 vaccination coverage and the booster dose among community-dwelling individuals with spinal cord injury and disease. Model 1 evaluated the potential characteristics impacting the uptake of COVID-19 vaccine full dose series (0 vs minimum 2 doses). Model 2 evaluated the potential characteristics impacting the uptake of the COVID-19 vaccine booster dose (2 doses only vs 3 or more doses). Odds ratios with 95% confidence interval, and *p*-values for corresponding characteristics displayed in the table.

Characteristics	Model 1: Uptake of COVID-19 Vaccine (0 Doses vs. Minimum 2 Doses)		Model 2: Uptake of COVID-19 Booster (2 Doses Only vs. 3+ Doses)	
	Odd Ratio (95% CI)	*p*-Value	Odd Ratio (95% CI)	*p*-Value
**Sex**				
Female	1.00 (0.80–1.25)	1.00	1.12 (0.90–1.38)	0.31
Male	REFERENCE			
**Age**				
44 and under	0.64 (0.46–0.90)	0.01	0.21 (0.15–0.29)	<0.01
45–64	1.05 (0.78–1.40)	0.77	0.40 (0.31–0.54)	<0.01
65+	REFERENCE			
**Injury Type**				
Traumatic Spinal Cord Injury (TSCI)	REFERENCE			
Non-Traumatic Spinal Cord Dysfunction (NTSCD)	1.29 (1.02–1.64)	0.03	1.54 (1.24–1.91)	<0.01
**Income Quintile**				
Q1 (low)	0.41 (0.26–0.64)	<0.01	0.39 (0.26–0.59)	<0.01
Q2	0.38 (0.25–0.57)	<0.01	0.67 (0.47–0.97)	0.03
Q3	0.56 (0.37–0.83)	<0.01	0.82 (0.58–1.16)	0.26
Q4	0.68 (0.46–1.00)	0.05	0.84 (0.61–1.18)	0.32
Q5 (high)	REFERENCE			
**Racialized and Newcomer Population Quintile**				
Q1 (low)	0.81 (0.56–1.17)	0.26	1.18 (0.84–1.65)	0.34
Q2	1.07 (0.74–1.55)	0.72	1.23 (0.88–1.71)	0.23
Q3	0.75 (0.53–1.06)	0.10	1.22 (0.87–1.71)	0.25
Q4	0.86 (0.62–1.18)	0.35	1.24 (0.92–1.69)	0.16
Q5 (high)	REFERENCE			
**Households and Dwellings Quintile**				
Q1 (low)	0.92 (0.61–1.39)	0.70	0.48 (0.32–0.72)	<0.01
Q2	0.99 (0.67–1.45)	0.95	0.49 (0.33–0.71)	<0.01
Q3	1.50 (1.03–2.18)	0.04	0.52 (0.36–0.75)	<0.01
Q4	1.17 (0.86–1.60)	0.31	0.51 (0.38–0.70)	<0.01
Q5 (high)	REFERENCE			
**Age and Labour Force Quintile**				
Q1 (low)	0.65 (0.45–0.92)	0.01	0.67 (0.48–0.92)	0.02
Q2	0.71 (0.51–1.01)	0.06	0.85 (0.62–1.18)	0.33
Q3	0.69 (0.49–0.98)	0.04	0.82 (0.60–1.14)	0.24
Q4	0.69 (0.49–0.96)	0.03	0.77 (0.57–1.06)	0.11
Q5 (high)	REFERENCE			
**Resource Utilization Bands (RUBs)**				
0	0.09 (0.06–0.13)	<0.01	0.55 (0.31–1.00)	0.05
1	1.05 (0.48–2.30)	0.91	0.34 (0.16–0.72)	<0.01
2	0.85 (0.55–1.32)	0.47	0.49 (0.32–0.76)	<0.01
3	1.18 (0.85–1.62)	0.33	0.61 (0.45–0.82)	<0.01
4	1.31 (0.90–1.91)	0.16	0.68 (0.49–0.95)	0.02
5	REFERENCE			
**Counts for Unique Medication(s)**				
0 *	0.43 (0.26–0.70)	<0.01	0.46 (0.30–0.72)	<0.01
1–9	0.68 (0.43–1.08)	0.10	0.75 (0.49–1.16)	0.20
10–19	0.81 (0.52–1.28)	0.37	0.62 (0.41–0.94)	0.02
≥20	REFERENCE			
**Previous Diagnosis of Mental Condition(s)**				
Yes	REFERENCE			
No	1.05 (0.81–1.37)	0.69	1.47 (1.14–1.90)	<0.01
**Number of Health Conditions**				
0–2	0.76 (0.53–1.10)	0.14	0.88 (0.63–1.23)	0.46
3–4	0.66 (0.49–0.89)	<0.01	0.97 (0.74–1.27)	0.80
5+	REFERENCE			

* Note: The number of unique medications were based on ODB reports only; medications not covered by ODB were not captured in this analysis. Model 1: The Hosmer–Lemeshow test yielded a chi-square statistic of 10.20 (df = 8, *p* = 0.2513), indicating that the model fits the data. Model 2: The Hosmer–Lemeshow test yielded a chi-square statistic of 12.63 (df = 8, *p* = 0.1251), indicating that the model fits the data.

## Data Availability

The dataset from this study is held securely in coded form at ICES. While legal data sharing agreements between ICES and data providers (e.g., healthcare organizations and government) prohibit ICES from making the dataset publicly available, access may be granted to those who meet pre-specified criteria for confidential access, available at www.ices.on.ca/DAS (accessed on 8 August 2024) (email: das@ices.on.ca). The full dataset creation plan and underlying analytic code are available from the authors upon request, understanding that the computer programs may rely upon coding templates or macros that are unique to ICES and are therefore either inaccessible or may require modification.

## Abbreviations

SCI/D	Spinal cord injury and disease
TSCI	Traumatic Spinal Cord Injury
NTSCD	Non-traumatic Spinal Cord Dysfunction
PHIPA	Personal Health Information Protection Act
DAD	Discharge Abstract Database
NRS	National Rehabilitation System
COVaxON	COVID-19 vaccination information system of Ontario
RPDB	Registered Persons Database
ODB	Ontario Drug Benefits Database
OHIP	Ontario Health Insurance Plan
ON-Marg	Ontario Marginalization Index
RUB	Resource Utilization Bands
